# MRI‐DTI Biomarkers Along the Continuum of Behavioral Variant Frontotemporal Dementia

**DOI:** 10.1111/ene.70438

**Published:** 2025-11-30

**Authors:** Marco Michelutti, Hans‐Jürgen Huppertz, Sarah Anderl‐Straub, Heiko Volkmann, Daniele Urso, Benedetta Tafuri, Salvatore Nigro, Paolo Manganotti, Angela Rosenbohm, Albert C. Ludolph, Markus Otto, Giancarlo Logroscino, Hans‐Peter Müller, Jan Kassubek

**Affiliations:** ^1^ Neurology Unit, Department of Medical, Surgical and Health Sciences University of Trieste Trieste Italy; ^2^ Department of Neurology University Hospital Ulm Ulm Germany; ^3^ Center for Neurodegenerative Diseases and the Aging Brain University of Bari Aldo Moro at Pia Fondazione “Card. G. Panico” Tricase Italy; ^4^ Swiss Epilepsy Clinic Klinik Lengg Zürich Switzerland; ^5^ Department of Engineering of Innovation University of Salento Lecce Italy; ^6^ Institute of Nanotechnology National Research Council (CNR‐NANOTEC) c/o Campus Ecotekne Lecce Italy; ^7^ Department of Neurology, University Hospital Halle Martin Luther University Halle (Saale) Germany; ^8^ Department of Diagnostic and Interventional Radiology University Hospital Ulm Ulm Germany; ^9^ Department of Nuclear Medicine University Hospital Ulm Ulm Germany

**Keywords:** ABV, ALS‐FTD, bvFTD, DTI, longitudinal analysis

## Abstract

**Background:**

We investigated whether diffusion tensor imaging (DTI) and atlas‐based volumetry (ABV) could track specific patterns of brain white matter (WM) microstructure and gray matter (GM) volumes in behavioral variant frontotemporal dementia (bvFTD) and amyotrophic lateral sclerosis with frontotemporal dementia (ALS‐FTD).

**Methods:**

MRI datasets from 65 bvFTD (including 19 with longitudinal MRI), 18ALS‐FTD, and 39 controls were analyzed. White matter fractional anisotropy (FA) differences were assessed using unbiased Whole Brain‐based Spatial Statistics (WBSS) and a hypothesis‐driven complementary approach consisting of Tract‐Wise FA Statistics (TFAS) in Tracts of Interest (TOIs) and ABV in Structures of Interest (SOIs). FA maps were correlated with disease severity (FTLD‐CDR sum of boxes). A random forest algorithm classified participants employing TOI and SOI data.

**Results:**

At baseline, both bvFTD and ALS‐FTD exhibited WM changes in several tracts including the uncinate fasciculi, tracts originating in the corpus callosum, and the inferior and superior longitudinal fasciculi. Atrophy was most pronounced in the frontal lobes and caudate nuclei. Longitudinally, bvFTD demonstrated an antero‐posterior spread of WM degeneration, particularly along the corpus callosum and inferior longitudinal fasciculus, with relatively modest cortical atrophy progression. Random forest analysis identified the most discriminative TOIs and SOIs including the uncinate fasciculus and the amygdala.

**Conclusions:**

Our findings demonstrate a similar pattern of structural and microstructural changes in bvFTD and ALS‐FTD, with a specific involvement of the corticospinal tract for ALS‐FTD, and support the utility of combined DTI and ABV in tracking disease progression across the FTLD spectrum.

## 1 Introduction

Frontotemporal lobar degeneration (FTLD) encompasses a group of neurodegenerative disorders that predominantly affect the frontal and temporal lobes of the brain, leading to changes in behavior, language, executive function, and motor abilities [1]. The most frequent clinical phenotype is the behavioral variant of frontotemporal dementia (bvFTD), characterized by personality changes and executive dysfunction [2]. Additionally, approximately 10%–15% of patients diagnosed with bvFTD exhibit motor neuron impairment, leading to a coexisting diagnosis of amyotrophic lateral sclerosis (ALS) [3]. Conversely, in patients with an initial diagnosis of ALS, the development of frontotemporal dementia characterized by cognitive and behavioral impairment has also been extensively documented (amyotrophic lateral sclerosis associated with frontotemporal dementia. ALS‐FTD) [4]. This has led to the conceptualization of an ALS–FTD continuum, where the combined ALS‐FTD syndrome has been suggested to be a phenotypical variant closer to bvFTD from the outset, rather than a late stage to which pure motor ALS cases progress [5]. This clinical overlap [6] is reflected at the neuropathological level, as both syndromes share a common hallmark, that is, the accumulation of TAR DNA‐binding protein (TDP‐43) [7] type A and B [8], whose aggregates underlie around 50% of bvFTD [9] (with tau pathology representing the second most frequent neuropathological correlate [10]) and the majority of ALS [11] cases. There is also overlap at the genetic level, where an expansion of the GGGGCC hexanucleotide repeat in the non‐coding intronic region of the *chromosome 9 open reading frame 72* gene (*C9orf72*) underlies up to 30% of the bvFTD [12, 13] and 40% of the familial ALS [12, 14] cases.

The combined syndrome of ALS‐FTD, which has been found to be driven by a *C9orf72* mutation in approximately 20%–30% of cases [15, 16], provides a shortcut for translating clinical observations into neuropathological insights, as there is a reliable correspondence between this clinical presentation and the presence of TDP‐43 aggregates [11, 16, 17, 18]. However, in sporadic bvFTD, TDP‐43 aggregates can only be confirmed post‐mortem, as there are currently no available biomarkers able to detect TDP‐43 in vivo. In the absence of a reliable biomarker capable of directly demonstrating the removal of TDP‐43 from the brain, clinical trials of potential disease‐modifying therapies in FTLD and ALS have relied on indirect measures, such as brain volumetry and indices of microstructural integrity. For this reason, elucidating the natural history of the progression of these changes becomes especially important, as it provides the basis for defining meaningful endpoints of efficacy in future clinical trials [19]. These endpoints would not be constrained by the availability of genetic or pathology‐specific markers, although it is acknowledged that sporadic and genetic cases may follow partially distinct trajectories of disease progression [20]. Instead, they offer clinically meaningful measures of disease progression that remain applicable to patients without a confirmed genetic or neuropathological status, as well as to trials of therapies that do not directly target a measurable molecular biomarker.

TDP‐43 aggregates are thought to spread trans‐synaptically, following the degree of functional [21] and structural [21] connectivity between regions that belong to the same brain functional networks rather than direct contiguity [22]. This constellation indicates Diffusion Tensor Imaging (DTI) as especially suited for the assessment of the progression of FTLD proteinopathies along white matter (WM) fiber bundles. Previous work from this group has applied DTI, a technique assessing microstructural WM integrity through water diffusion. in studies on TDP‐43‐related bvFTD [23] and ALS [24]. In these, fractional anisotropy (FA), a summary measure of microstructural integrity, was proved to be the most reliable and standardized metric to track disease progression [23, 25]. For this reason, the primary aim of the current study was to characterize changes in both GM and WM integrity in bvFTD using a multiparametric magnetic resonance imaging (MRI) approach, combining DTI and unbiased atlas‐based volumetry (ABV) [26, 27] and to investigate their progression over time.

Furthermore, we expected to clarify the positioning of ALS‐FTD within the TDP‐43 clinico‐pathological continuum. Establishing whether it represents a phenotype more closely aligned with bvFTD or an “extra‐motor” evolution of ALS carries important implications for patient stratification and clinical trial design.

Specifically, we wanted to: (I) identify whole brain‐based DTI alterations. WM tract‐of‐interest (TOI)‐based FA changes, and volumetric differences in structures of interest (SOIs) in bvFTD and ALS‐FTD; (II) employ a random forest algorithm to identify the imaging features most discriminative for patient classification; (III) evaluate longitudinal changes in DTI and ABV metrics in a subset of bvFTD patients with an available follow‐up MRI scan; and (IV) examine associations between clinical progression and DTI measures.

## 2 Methods

### 2.1 Subjects and MRI Acquisition

Sixty‐five patients with a diagnosis of bvFTD and 18 patients with a diagnosis of ALS‐FTD were retrospectively included in the study. Controls (*n* = 39) included 10 healthy subjects and 29 subjects with subjective cognitive decline (SCD) [28] and negative CSF biomarkers for neurodegeneration amyloid and tau pathology. For clarity, the term “controls” is used consistently throughout the manuscript to collectively refer to both healthy volunteers and individuals with subjective cognitive decline (SCD) who had negative CSF biomarkers. Diagnoses of bvFTD were formulated according to established diagnostic criteria [2].

Patients in the ALS‐FTD group required a confirmed diagnosis of ALS together with behavioral/cognitive changes consistent with bvFTD [2]. That is, at least three core behavioral/cognitive symptoms, or at least two such symptoms in combination with loss of insight and/or psychotic features [4], as per ALS‐FTSD (amyotrophic lateral sclerosis—frontotemporal spectrum disorder) revised criteria [4]. Furthermore, all patients in the ALS‐FTD group showed evidence of progressive deterioration of behavior and/or cognition that compromised functional independence in activities of daily living, as reflected by an FTLD‐CDR global score ≥ 1. A comprehensive set of cognitive and neuropsychological tests was administered to assess patients at baseline and to support the diagnostic process (Appendix S1).

Data acquisition was performed at two study sites: Ulm, Germany, (cohort “A”) and Tricase, Italy (cohort “B”). The study was approved by the local ethics committees (Ethics Committee of the University of Ulm, reference 39/11 and Institutional Review Board of Azienda Sanitaria Locale Lecce, report n. 6, 25 July 2017, respectively) and written informed consent was obtained from each participant or the primary caregiver in accordance with the Declaration of Helsinki. The sum of boxes of the FTLD Clinical Dementia Rating (CDR) as a validated score for measuring progression of functional decline [29] (minimum possible score: 0; maximum possible score: 24) was available for all patients.

In cohort A, 52 bvFTD, 14 ALS‐FTD, and 14 controls underwent 3.0T MRI (Siemens Allegra), while 2 bvFTD, 4 ALS‐FTD, and 1 control underwent a 1.5T MRI (Siemens Symphony); 19 bvFTD patients obtained a follow‐up scan after 12 months (on average). In cohort B, 11 bvFTD and 24 controls underwent 3.0T MRI (Philips Ingenia).

DTI data were acquired using two protocols. Cohort A: 31 gradient directions (GD), b = 1000 s/mm^2^, echo time (TE) = 88 ms, repetition time (TR) = 11,100 ms, voxel size 2.0 × 2.0 × 2.0 mm^3^, Cohort B: 65 GD, b = 1000 s/mm^2^, TE = 85 ms, TR = 6852 ms, voxel size 2.5 × 2.5 × 2.5 mm^3^.

For cohort A, structural T1‐weighted data included 144 sagittal slices (1.2 mm thickness), 1.0 × 1.0 mm^2^ in‐plane resolution in a 256 × 248 matrix, TE = 4.2 ms and TR = 1640 ms; for cohort B, T1‐weighted data included 200 slices (matrix 256 × 256), voxel size = 1 mm^3^ isotropic, TR = 820 ms, TE = 3.8 ms. The features of the two cohorts are summarized in Table S1.

### 2.2 Microstructural MRI Analysis

#### 2.2.1 Standardized Data Pre‐Processing

The DTI analysis software *Tensor Imaging and Fiber Tracking* (TIFT) [30, 31] was used for pre/post‐ processing and statistical analysis, following established methodological guidelines previously described in detail [25, 30, 32, 33]. In short, after motion correction, spatial normalization to the MNI stereotaxic standard space was performed iteratively using study‐specific templates. All data were assessed for completeness, and, according to an established quality control protocol [34], corrupted gradient directions (GD) as well as motion artifacts were excluded from further analysis prior to correction of eddy current‐induced geometric distortions. Data were interpolated onto a 1 mm isotropic voxel grid for all further analyses [33]. An isotropic three‐dimensional 8 mm full‐width at half‐maximum Gaussian smoothing filter was applied to the individual normalized FA maps, providing a good balance between sensitivity and specificity. FA maps were corrected for age using regression models based on datasets from 15 and 24 controls, separately for the two contributing centers. Subsequently, FA maps of patients with bvFTD and controls were harmonized for each center by applying respective 3D correction matrices (linear first‐order correction). These 3D correction matrices were derived as linear adjustments based on differences in the DTI scans of controls from each center. No residual site effects could be detected after inter‐center correction. While the control group had a lower proportion of male participants compared to the patient groups, we did not apply a correction for sex, as there is no established evidence of sex‐related differences in DTI or ICV‐corrected ABV metrics in healthy individuals. Even if present, any observed sex effects in patient groups would be more likely attributable to disease‐related pathology than to sex itself, particularly given the modest sample size. The same protocol was applied to mean diffusivity (MD), an additional measure of membrane integrity (Figure S4A,B).

#### 2.2.2 Definition of Tract Structures

To apply group‐based fiber tracking (FT) algorithms [35], an averaged DTI dataset was generated from 24 control datasets with an identical acquisition protocol, to avoid bias from pathology‐induced alterations. This averaged DTI dataset was then utilized to identify pathways for defined TOIs. A previous study [23], translating a neuropathological staging hypothesis for TDP43‐bvFTD [36] in vivo, was used as a reference for TOI definition.

A seed‐to‐target approach was used [24, 32] in which all fiber tracts (FTs) originating in the seed regions and terminating in the target regions of the respective pathway defined the corresponding TOI.

The technique of tract‐wise fractional anisotropy statistics (TFAS) [31] was employed to quantify the tractography results using the TOIs. The FA values of the specific tracts were arithmetically averaged for each stereotaxically normalized DTI dataset of each subject. The following TOIs were thus defined for the bvFTD and ALS‐FTD groups: left and right uncinate fasciculus (UF), genu, section II, III, IV and splenium of the corpus callosum (CC), left and right superior longitudinal fasciculus (SLF), left and right inferior longitudinal fasciculus (ILF), inferior fronto‐occipitalis fasciculus (IFOF), cingulum, pons/brainstem, anterior thalamic radiation, corticostriatal tract, corticospinal tract (CST), optic radiation, fornix, left and right tapetum. The same protocol was applied to extract TOI‐based MD values (Figure S4A,B).

### 2.3 Data Post‐Processing

#### 2.3.1 Atlas‐Based Volumetry

The T1‐weighted data were processed using MATLAB (version R2014b, The Mathworks, USA) and the Statistical Parametric Mapping 12 (SPM12) software (Wellcome Trust Center for Neuroimaging, London, UK, www.fil.ion.ucl.ac.uk/spm), following a standardized processing pipeline for atlas‐based volumetry (ABV) [27]. The processing steps included: (i) segmentation into GM, WM, and cerebrospinal fluid (CSF) compartments, (ii) stereotaxic normalization into MNI space, and (iii) volumetric assessment using voxel‐by‐voxel multiplication and subsequent integration of normalized modulated component images (GM, WM, or CSF) with predefined masks from various brain atlases. All volumetric results were linearly standardized to the mean intracranial volume (ICV) of controls. Group‐level differences were tested for significance after correction for multiple comparisons.

A series of standard cortical and subcortical SOIs were chosen for ABV analysis [27]. These were the cerebrum GM and WM, frontal lobes, temporal lobes, parietal lobes, occipital lobes, insulae, cerebellum, brainstem, hippocampus, amygdala, caudate, putamen, and thalamus (Figure S4C).

#### 2.3.2 Whole Brain–Based Voxel‐Wise Statistics at the Group Level

Whole brain–based spatial statistics (WBSS) [37] was used to calculate cross‐sectional and longitudinal differences in age and scanner‐corrected FA and MD maps. Statistical comparisons between the patients with bvFTD (*n* = 65), ALS‐FTD (*n* = 18), and 39 controls were performed voxel‐wise using Welch's test, with the FA threshold set at 0.2 [37, 38]. Statistical results were corrected for multiple comparisons using the false discovery rate (FDR) algorithm at *p* < 0.05 [39]. Type 1 error was further reduced by applying a cluster size threshold of 256 mm^3^.

#### 2.3.3 Tract‐Of‐Interest Wise Comparison at the Group Level

Age and scanner‐corrected FA and MD maps from baseline scans were used to calculate mean FA and MD values for the respective TOIs. Cross‐sectional and longitudinal (*n* = 19, bvFTD group) comparisons of mean FA and MD values between patients and controls were then performed.

#### 2.3.4 Structure‐Of‐Interest Wise Comparison at the Group Level

For the detection of volumetric alterations, group‐level cross‐sectional and longitudinal (*n* = 19, bvFTD group) differences in ABV were assessed for statistical significance following correction for multiple comparisons in each SOI, using age as a covariate.

#### 2.3.5 Cross‐Sectional Correlation of FA Maps and ABV Data to Clinical Scores

The age‐corrected and inter‐center harmonized FA maps from 65 patients with bvFTD and 18 patients with ALS‐FTD were voxel‐wise correlated to the sum of boxes of the FTLD‐CDR scores (FDR‐ and cluster‐size‐corrected). For the correlation analyses spherical regions of interest (ROIs) were automatically placed bi‐hemispherically within the peak result clusters of WBSS.

Furthermore, a partial correlation was performed between the ICV‐standardized ABV data for each SOI and the sum of boxes of the FTLD‐CDR score (corrected at *p* < 0.05), using age as a covariate.

### 2.4 Classification by Machine Learning: Random Forest Model

For the selection of the most important features to distinguish patients with bvFTD and ALS‐FTD from controls, two Random Forest algorithms were implemented. Implementation, training, validation, feature selection, and hierarchical ordering were used as reported in a previous study [40, 41, 42, 43, 44, 45].

This model allows for an understanding of the hierarchical order of the selected features by means of the Gini importance. As the model was trained using FA values from the TOIs employed in the TFAS analyses along with SOIs employed in the ABV analysis, its application was instrumental in identifying key SOIs and TOIs that significantly contributed to the classification task [42, 43, 44]. Furthermore, random forest models have been used successfully in previous studies with a similar data structure combining DTI and T1‐weighted data [40, 41].

For model development, the dataset was divided into 80% training and 20% validation cohorts. Two controls had to be excluded from the analysis as their volumetric data were compromised by artifacts. The training cohort included 30 controls, 52 patients with bvFTD and 14 patients with ALS‐FTD. For validation, the remaining 7 controls, 13 bvFTD patients, and 4 ALS‐FTD patients were used. To reduce the risk of overfitting because of the limited sample size, a 5‐fold cross‐validation was applied [46]. This means that each model for a defined feature selection was implemented five times: in each iteration, training was done on four folds. This iteration was repeated until each fold had served once as the validation set.

The Random Forest classifiers were implemented using the Scikit‐learn library [45]. Key hyperparameters were as follows: number of trees: 100; splitting criterion: Gini index; tree depth: 4. Additionally, the minimum number of samples required to split a node was set to 2 and the minimum number of samples required at a leaf node was set to 1. For each split, a random subset of features (square root of the total number) was considered (max_features = “sqrt”) and bootstrap sampling was enabled. All models were trained using a fixed random state to ensure reproducibility. Feature selection was performed iteratively by removing the least important features based on the Gini index, retaining only those that maintained or improved classification accuracy [42, 46].

## 3 Results

### 3.1 Demographic Data

The patients' demographic and clinical features are summarized in Table 1.

**TABLE 1:** Demographic and clinical features of participants.

	bvFTD (*n* = 65)	ALS‐FTD (*n* = 18)	Controls (*n* = 39)	*p*
Age	63.8 ± 10.1	64.0 ± 9.7	57.9 ± 11.6	ns
Sex, males %	58.5%	44.4%	17.9%	
Disease duration (months)	48.0 ± 35.2	35.1 ± 19.1		
Education (years)	13.0 ± 3.5	13.5 ± 3.8	13.0 ± 2	ns
MMSE	24.5 ± 4.9	25.0 ± 3.1	27.5 ± 1.2	< 0.001
FTLD‐CDR sum of boxes	5.1 ± 3.6	6.3 ± 5.8		

*Note:* This table shows the demographic and clinical features in the bvFTD, ALS‐FTD and control groups. Age, MMSE and FTLD‐CDR sum of boxes are shown as mean ± standard deviation; sex is shown as % of males on the total number of subjects in each group. *p* values are shown when the differences are significant.

Abbreviations: ALS‐FTD, amyotrophic lateral sclerosis associated with frontotemporal dementia; bvFTD, behavioral variant of frontotemporal dementia; TOIs, tracts of interest; ns, nonsignificant.

The patients' mean age (bvFTD, 63.83 ± 10.05; ALS‐FTD, 64 ± 9.76) was not significantly different from that of the controls (57.91 ± 11.61). Thirty‐eight/65 and 8/18 of the subjects were male in the bvFTD and in the ALS‐FTD groups, respectively, while 7/39 of the subjects were male in the control group. The subjects were significantly different in mean MMSE scores (controls, 27.54 ± 1.21; bvFTD, 24.5 ± 4.92; ALS‐FTD, 25 ± 3.10; *p* < 0.001). In addition, the two control subgroups showed a statistically significant difference in mean MMSE scores (SCD: 27.67 ± 1.27; HC: 29.67 ± 0.50; *p* < 0.001). However, this difference was not considered clinically meaningful, as all individual scores remained above the standard cutoff of 24 for cognitive impairment. For DTI‐based FA analyses as well as for GM volumetric analyses, no significant differences between SCD and healthy controls could be detected (Figure S1, Tables S2 and S3). The mean sum of boxes of the FTLD‐CDR scores was 5.10 ± 3.64 in the bvFTD group and 6.39 ± 5.85 in the ALS‐FTD group, respectively.

### 3.2 WBSS of Cross‐Sectional Microstructural Differences

**FIGURE 1:**
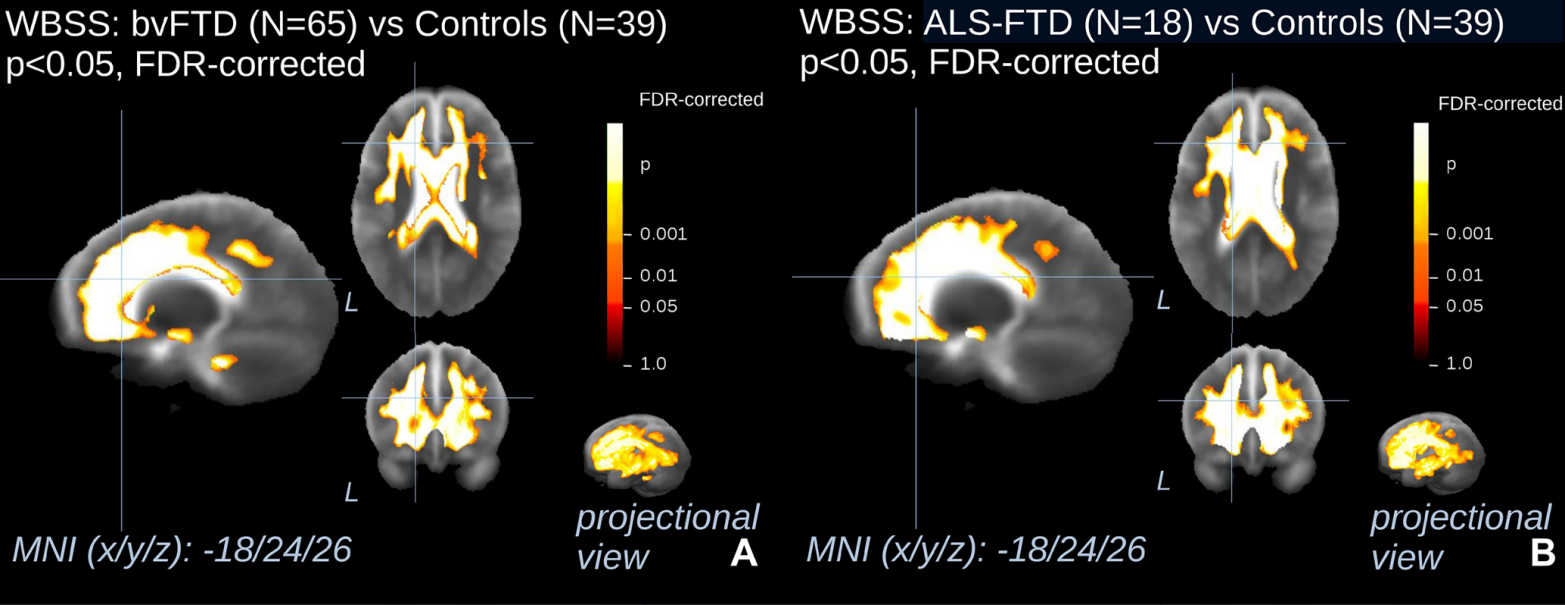
Whole‐brain‐based spatial statistics for cross‐sectional comparison of FA maps of patients with bvFTD and patients with ALS‐FTD versus controls. This figure shows slice‐wise and projectional views of cross‐sectional differences in baseline fractional anisotropy (FA) in 65 patients with behavioral variant of frontotemporal dementia (bvFTD, A) and 18 patients with amyotrophic lateral sclerosis associated with frontotemporal dementia (ALS‐FTD, B) versus 39 controls. A projectional overlay reveals all clusters in a pseudo‐3D view (bottom right). Significance level is coded according to the color bar. Corrections for multiple comparisons were made with the false discovery rate at *p* < 0.05 and a cluster‐size approach. Details of the corresponding clusters are shown in Table S4. ALS‐FTD, amyotrophic lateral sclerosis associated with frontotemporal dementia; bvFTD, behavioral variant of frontotemporal dementia; MNI, Montreal Neurological Institute; WBSS, whole brain spatial statistics.

The group differences in WM FA values demonstrated a significant decrease along a widespread cluster spanning the WM fibers connecting the frontal and temporal lobes, such as the UF, and the temporal lobes with posterior regions of the brain, such as the SLF and the ILF, as well as the CC. This pattern was shared by both the patients with bvFTD and those with ALS‐FTD, as opposed to controls (Figure 1A,B, Table S4).

A comparison of the spatial extent of these clusters between the two pathological groups revealed an additional cluster in the upper CST in ALS‐FTD patients which was not observed in the bvFTD group (Figure S2A–D).

To validate the differences in FA between groups, an additional analysis was performed on MD maps. Unbiased WBSS results for MD differences were largely comparable to those obtained for FA differences in both bvFTD and ALS‐FTD relative to controls (Figure S3A–C).

### 3.3 Cross‐Sectional Microstructural Differences in the White Matter TOIs at Group‐Level

Significant group differences in mean FA (and MD) of the TOIs between bvFTD and ALS‐FTD as opposed to controls were largely identical (Table 2 for FA differences; Table S5 for MD differences).

**TABLE 2:** Cross‐sectional fractional anisotropy (FA) differences in the white matter TOIs at group‐level.

Tract of interest (TOI) analysis	bvFTD				ALS‐FTD					Controls	
	Mean	Standard deviation	*z* score bvFTD vs Controls	*p*	Mean	Standard deviation	*z* score ALS‐FTD vs Controls	*p*	*z* score ALS‐FTD vs bvFTD	Mean	Standard deviation
Left uncinate fasciculus	0.26	0.03	−1.78	< 0.001	0.25	0.03	−2.18	< 0.001	−0.27	0.29	0.02
Right uncinate fasciculus	0.26	0.03	−1.12	< 0.001	0.25	0.02	−1.57	< 0.001	−0.34	0.28	0.02
Genu of the corpus callosum	0.29	0.07	−1.32	< 0.001	0.26	0.06	−1.86	< 0.001	−0.43	0.35	0.05
Splenium of the corpus callosum	0.32	0.06	−0.88	0.001	0.31	0.05	−0.98	0.011	−0.09	0.36	0.05
Section II of the corpus callosum	0.26	0.03	−0.87	0.003	0.25	0.02	−1.31	< 0.001	−0.33	0.28	0.02
Section III of the corpus callosum	0.29	0.03	−0.46	ns	0.27	0.03	−0.88	0.010	−0.44	0.30	0.04
Section IV of the corpus callosum	0.30	0.06	−1.01	< 0.001	0.29	0.03	−1.20	< 0.001	−0.18	0.35	0.05
Left superior longitudinal fasciculus	0.33	0.05	−0.73	0.029	0.32	0.05	−0.99	ns	−0.16	0.35	0.03
Right superior longitudinal fasciculus	0.36	0.06	−0.65	0.048	0.38	0.05	−0.25	ns	0.22	0.38	0.03
Left inferior longitudinal fasciculus	0.29	0.03	−0.84	0.001	0.29	0.03	−1.10	0.011	−0.24	0.32	0.03
Right inferior longitudinal fasciculus	0.30	0.05	−0.52	ns	0.29	0.04	−0.83	ns	−0.26	0.32	0.04
Inferior fronto‐occipitalis fasciculus	0.27	0.03	−1.50	< 0.001	0.26	0.02	−1.92	< 0.001	−0.32	0.30	0.02
Cingulum	0.32	0.06	−1.48	< 0.001	0.30	0.05	−2.02	< 0.001	−0.37	0.38	0.04
Pons/brainstem	0.34	0.04	−0.90	0.003	0.33	0.03	−1.30	0.003	−0.29	0.36	0.03
Anterior thalamic radiation	0.26	0.03	−1.16	< 0.001	0.25	0.03	−1.61	< 0.001	−0.38	0.29	0.02
Corticostriatal projections	0.25	0.04	−1.48	< 0.001	0.23	0.03	−2.26	< 0.001	−0.57	0.29	0.03
Corticospinal tract	0.35	0.04	−0.71	0.029	0.34	0.03	−1.07	0.015	−0.25	0.37	0.03
Optic radiation	0.34	0.05	−0.98	< 0.001	0.34	0.05	−1.03	0.043	−0.04	0.37	0.04
Fornix	0.26	0.02	−0.43	0.042	0.26	0.03	−0.47	ns	−0.03	0.27	0.02
Left tapetum	0.31	0.05	−0.84	0.003	0.30	0.05	−0.92	ns	−0.06	0.34	0.04
Right tapetum	0.35	0.06	−0.66	0.025	0.37	0.06	−0.14	ns	0.40	0.38	0.04

*Note:* This table shows the differences in mean fractional anisotropy (FA) values at baseline between 65 patients with bvFTD, 18 patients with ALS‐FTD, and 39 controls. *p* values are reported if the difference between the mean volumetric and FA values of controls and patients with bvFTD and ALS‐FTD, respectively, was significant (*p* < 0.05, corrected for multiple comparisons). *z*‐scores were calculated as the difference between the mean of the groups, divided by the pooled standard deviations of the groups.

Abbreviations: ALS‐FTD: amyotrophic lateral sclerosis associated with frontotemporal dementia; bvFTD: behavioral variant of frontotemporal dementia; ns: non‐significant; St. Dev: Standard Deviation; TOIs: Tracts of Interest.

### 3.4 Cross‐Sectional Structural Differences in the Gray Matter SOIs at Group‐Level

In the ABV analysis, both bvFTD and ALS‐FTD patients exhibited multiple significant volumetric reductions compared to controls. These results are summarized in Table 3A.

**TABLE 3A:** Cross‐sectional volumetric differences in the SOIs at group‐level.

ABV analysis	Controls		bvFTD		ALS‐FTD		*z*‐scores			Group comparison		
	Mean	SD	Mean	SD	Mean	SD	bvFTD vs Controls	ALS‐FTD vs Controls	ALS‐FTD vs bvFTD	bvFTD vs Controls	ALS‐FTD vs Controls	ALS‐FTD vs bvFTD
Cerebrum GM	533.7	55.0	450.1	60.5	417.2	48.7	−1.5	−2.1	−0.6	< 0.001	< 0.001	ns
Cerebrum WM	389.5	28.8	379.0	38.4	369.8	18.0	−0.4	−0.7	−0.3	ns	0.002	ns
Frontal lobe R	151.6	10.8	131.9	16.2	121.7	10.8	−1.8	−2.8	−1.0	< 0.001	< 0.001	0.022
Frontal lobe L	153.0	11.3	132.2	16.8	123.0	11.0	−1.9	−2.7	−0.8	< 0.001	< 0.001	0.039
Temporal lobe R	92.3	6.1	83.9	10.0	78.6	6.4	−1.4	−2.3	−0.9	< 0.001	< 0.001	0.039
Temporal lobe L	93.7	6.7	84.8	7.7	80.3	6.9	−1.4	−2.1	−0.7	< 0.001	< 0.001	ns
Parietal lobe R	82.8	5.4	75.9	6.7	74.0	6.5	−1.3	−1.7	−0.4	< 0.001	< 0.001	ns
Parietal lobe L	86.1	6.2	77.1	6.9	76.1	7.4	−1.5	−1.7	−0.2	< 0.001	< 0.001	ns
Occipital lobe R	62.1	4.7	57.8	5.8	55.6	5.6	−0.9	−1.4	−0.5	< 0.001	< 0.001	ns
Occipital lobe L	60.0	4.9	55.7	6.1	53.4	5.9	−0.9	−1.4	−0.5	< 0.001	< 0.001	ns
Insula R	8.1	0.8	7.0	1.0	6.5	0.8	−1.4	−2.2	−0.8	< 0.001	< 0.001	ns
Insula L	8.4	0.7	7.2	1.0	6.8	0.7	−1.6	−2.3	−0.7	< 0.001	< 0.001	ns
Cerebellum	113.3	10.4	105.7	10.4	100.4	8.0	−0.8	−1.3	−0.5	< 0.001	< 0.001	ns
Brainstem	30.1	2.5	29.4	2.6	27.7	1.4	−0.3	−1.0	−0.6	ns	< 0.001	0.012
Hippocampus R	3.4	0.3	2.9	0.5	2.5	0.4	−1.6	−2.9	−1.3	< 0.001	< 0.001	0.012
Hippocampus L	3.2	0.3	2.8	0.4	2.4	0.4	−1.5	−2.6	−1.1	< 0.001	< 0.001	0.022
Amygdala R	1.9	0.2	1.6	0.3	1.4	0.2	−1.7	−2.8	−1.2	< 0.001	< 0.001	0.012
Amygdala L	1.7	0.2	1.5	0.2	1.3	0.2	−1.7	−2.7	−1.0	< 0.001	< 0.001	0.039
Caudate R	2.2	0.2	1.6	0.5	1.3	0.5	−1.9	−3.1	−1.2	< 0.001	< 0.001	ns
Caudate L	2.3	0.3	1.7	0.6	1.5	0.5	−1.9	−2.6	−0.7	< 0.001	< 0.001	ns
Putamen R	3.2	0.5	2.6	0.6	2.4	0.3	−1.2	−1.6	−0.4	< 0.001	< 0.001	ns
Putamen L	3.3	0.5	2.5	0.6	2.4	0.5	−1.4	−1.7	−0.3	< 0.001	< 0.001	ns
Thalamus R	6.0	0.6	5.1	0.6	4.7	0.6	−1.6	−2.2	−0.6	< 0.001	< 0.001	ns
Thalamus L	6.3	0.7	5.3	0.6	4.9	0.6	−1.5	−2.1	−0.6	< 0.001	< 0.001	ns

*Note:* This table presents cross‐sectional and longitudinal differences in SOI volumes (mL). For the cross‐sectional analysis, the results are shown for 65 patients with bvFTD and 18 patients with ALS‐FTD versus 39 controls (Table 3A). For the longitudinal analysis (Table 3B), it shows volume changes in 19 patients with bvFTD from baseline to 1‐year follow‐up, compared with controls (*n* = 39), both as *z*‐scores and as % of volumetric reduction from baseline to follow‐up. Mean and standard deviation are reported for each group. *z*‐scores were calculated as the difference between the mean of the groups, divided by the pooled standard deviations of the groups. FDR‐corrected *p* values are reported if significant. A 2‐color scale was used to rank the *z*‐scores: light‐orange is used for −1 < z score <0; orange is used for −2 < z score < −1; red is used for z score < −2; light blue is used for z score > 0.

Abbreviations: ABV, atlas‐based‐volumetry; ALS‐FTD, amyotrophic lateral sclerosis associated with frontotemporal dementia; bvFTD, behavioral variant of frontotemporal dementia; FU, follow‐up; GM, gray matter; ns, nonsignificant; SD, standard deviation; SOIs, structures of interest; WM, white matter.

### 3.5 Longitudinal Differences for WBSS and TOI‐Based FA Values

**FIGURE 2:**
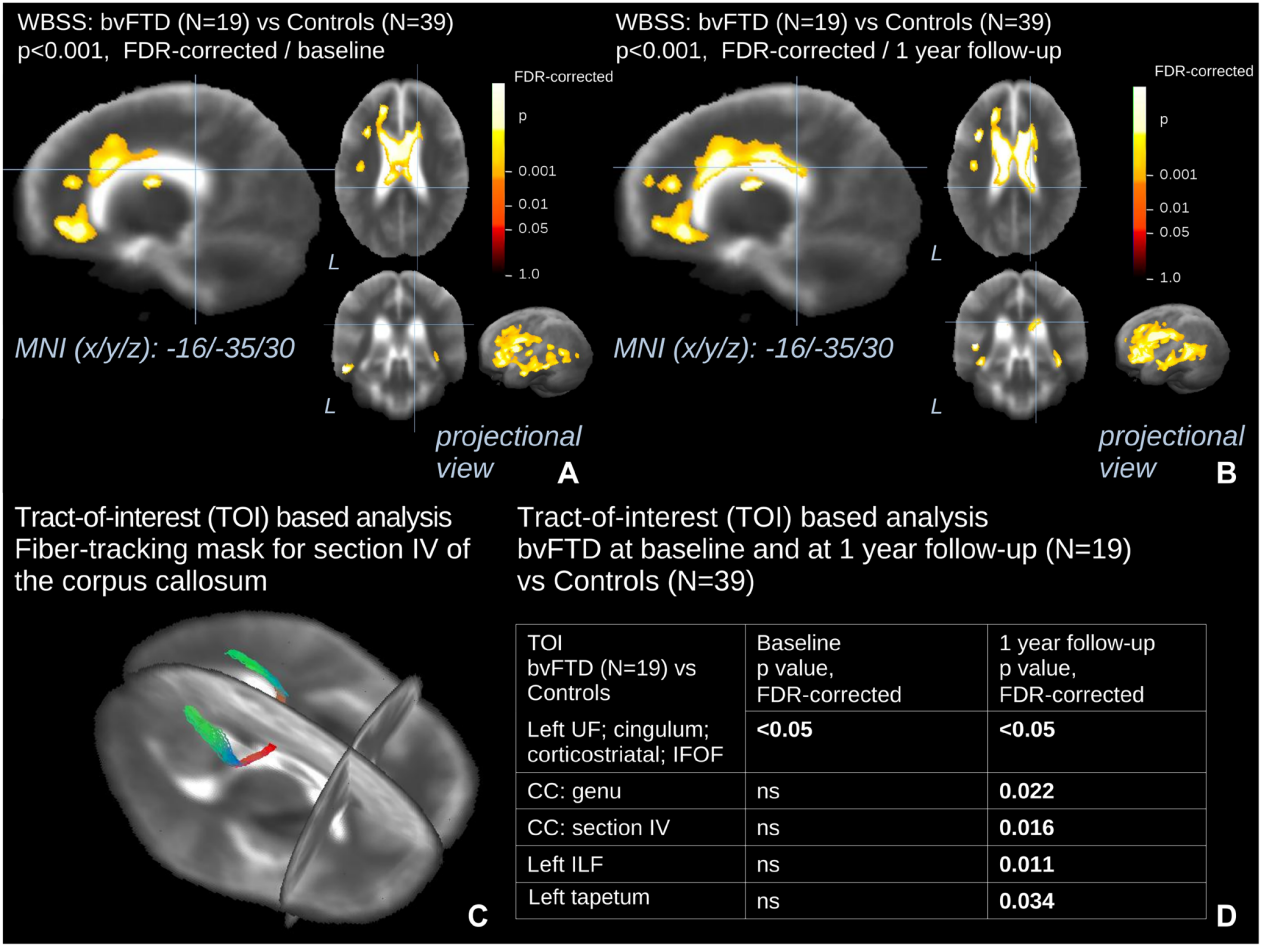
Whole‐brain‐based spatial statistics (WBSS) and tract‐of‐interest (TOI)‐based statistics for the longitudinal comparison of FA maps of patients with bvFTD versus controls. This figure shows slice‐wise and projectional views of WBSS (A, B) and TOI‐based (C, D) longitudinal differences in FA in 19 bvFTD patients from baseline to 1 year follow‐up versus 39 controls. The slices on the 3 axes are centered in the main cluster identified to show additional white matter involvement over the 1‐year long follow‐up, for the purpose of representation. A projectional overlay reveals all clusters in a pseudo‐3D view (bottom right). Significance level is coded according to the color bar. Corrections for multiple comparisons were made with the false discovery rate at *p* < 0.05 and a cluster‐size approach. Details of the corresponding clusters at baseline and at 1‐year follow‐up are shown in Table S5. In the lower section (C) a 3D representation of the tract where the difference in mean FA values had the higher degree of significance vs. controls is shown. *p* values for the corresponding TOI‐based statistics are displayed in the (D) quadrant (TOIs are displayed only when significant either at baseline or at 1 year follow‐up). bvFTD, behavioral variant of frontotemporal dementia; MNI, Montreal Neurological Institute; TOI, tract of interest; WBSS, whole brain spatial statistics.

For the longitudinal WBSS analysis, an increase in the size of all the clusters could be observed in the 19 patients with bvFTD versus controls (Figure 2A,B, Table S6). A longitudinal antero‐posterior progression of WM degeneration could be observed along the WM of the CC and frontal lobes as well as in the posterior sections of temporal WM. These significant longitudinal alterations were grosso modo consistent with the findings observed in the bvFTD‐related TOIs (Figure 2C,D).

### 3.6 Longitudinal Differences in SOI‐Based ABV Analysis

In the ABV analysis, the 19 bvFTD patients with follow‐up exhibited significant volumetric reductions compared to controls (Table 3B).

**TABLE 3B:** Longitudinal FA differences in the SOIs at group level for patients with bvFTD.

ABV analysis	Controls		bvFTD (baseline)		bvFTD (FU)		*z*‐scores		Volume changes (%)	Group comparison (against controls)	
	Mean	SD	Mean	SD	Mean	SD				*p*	
Cerebrum GM	533.7	55.0	426.5	63.9	406.3	67.4	−1.6	−2.0	−4.74	0.002	< 0.001
Cerebrum WM	389.5	28.8	389.0	51.1	392.0	60.2	0.3	0.3	+0.77	ns	ns
Frontal lobe R	151.6	10.8	131.3	19.6	129.2	23.0	−1.0	−1.2	−1.6	0.018	0.029
Frontal lobe L	153.0	11.3	130.4	21.3	127.4	25.0	−1.2	−1.4	−2.3	0.025	0.016
Temporal lobe R	92.3	6.1	82.1	13.4	80.3	14.0	−0.8	−1.0	−2.19	ns	ns
Temporal lobe L	93.7	6.7	81.6	10.6	79.1	11.7	−1.0	−1.4	−3.06	0.018	0.007
Parietal lobe R	82.8	5.4	74.8	9.6	73.1	10.0	−0.6	−0.9	−2.27	ns	0.041
Parietal lobe L	86.1	6.2	75.2	10.0	73.7	11.3	−1.0	−1.2	−1.99	0.024	0.015
Occipital lobe R	62.1	4.7	55.4	8.5	54.2	8.0	−0.8	−1.0	−2.17	ns	0.034
Occipital lobe L	60.0	4.9	53.3	8.4	52.3	7.7	−0.8	−1.0	−1.88	ns	0.034
Insula R	8.1	0.8	6.8	1.1	6.5	1.2	−1.3	−1.7	−4.41	0.011	0.002
Insula L	8.4	0.7	7.0	1.0	6.6	1.1	−1.6	−2.1	−5.71	0.004	0.001
Cerebellum	113.3	10.4	102.3	10.8	98.5	12.0	−0.7	−1.1	−3.71	ns	0.01
Brainstem	30.1	2.5	30.0	2.7	29.0	2.5	0.3	0.0	−3.33	ns	ns
Hippocampus R	3.4	0.3	2.9	0.5	2.8	0.5	−1.1	−1.5	−3.45	0.042	0.01
Hippocampus L	3.2	0.3	2.7	0.4	2.6	0.4	−1.5	−1.9	−3.7	0.010	0.002
Amygdala R	1.9	0.2	1.6	0.3	1.5	0.3	−1.6	−1.9	−6.25	0.006	0.001
Amygdala L	1.7	0.2	1.4	0.2	1.4	0.2	−1.7	−2.1	0.0	0.004	< 0.001
Caudate R	2.2	0.2	1.7	0.5	1.5	0.5	−1.4	−2.0	−11.76	0.018	0.002
Caudate L	2.3	0.3	1.7	0.6	1.6	0.6	−1.6	−2.3	−5.88	0.018	0.002
Putamen R	3.2	0.5	2.5	0.5	2.3	0.5	−1.1	−1.6	−8.0	0.005	< 0.001
Putamen L	3.3	0.5	2.5	0.6	2.1	0.6	−1.3	−1.9	−16.0	0.004	< 0.001
Thalamus R	6.0	0.6	5.1	0.7	5.0	0.7	−1.1	−1.3	−1.96	0.007	0.002
Thalamus L	6.3	0.7	5.1	0.8	5.0	0.8	−1.4	−1.7	−1.96	0.003	< 0.001

*Note:* See Table 3A for abbreviations and description.

### 3.7 Correlation of Voxel‐Wise FA Values With FTLD‐CDR Sum of Boxes

**FIGURE 3:**
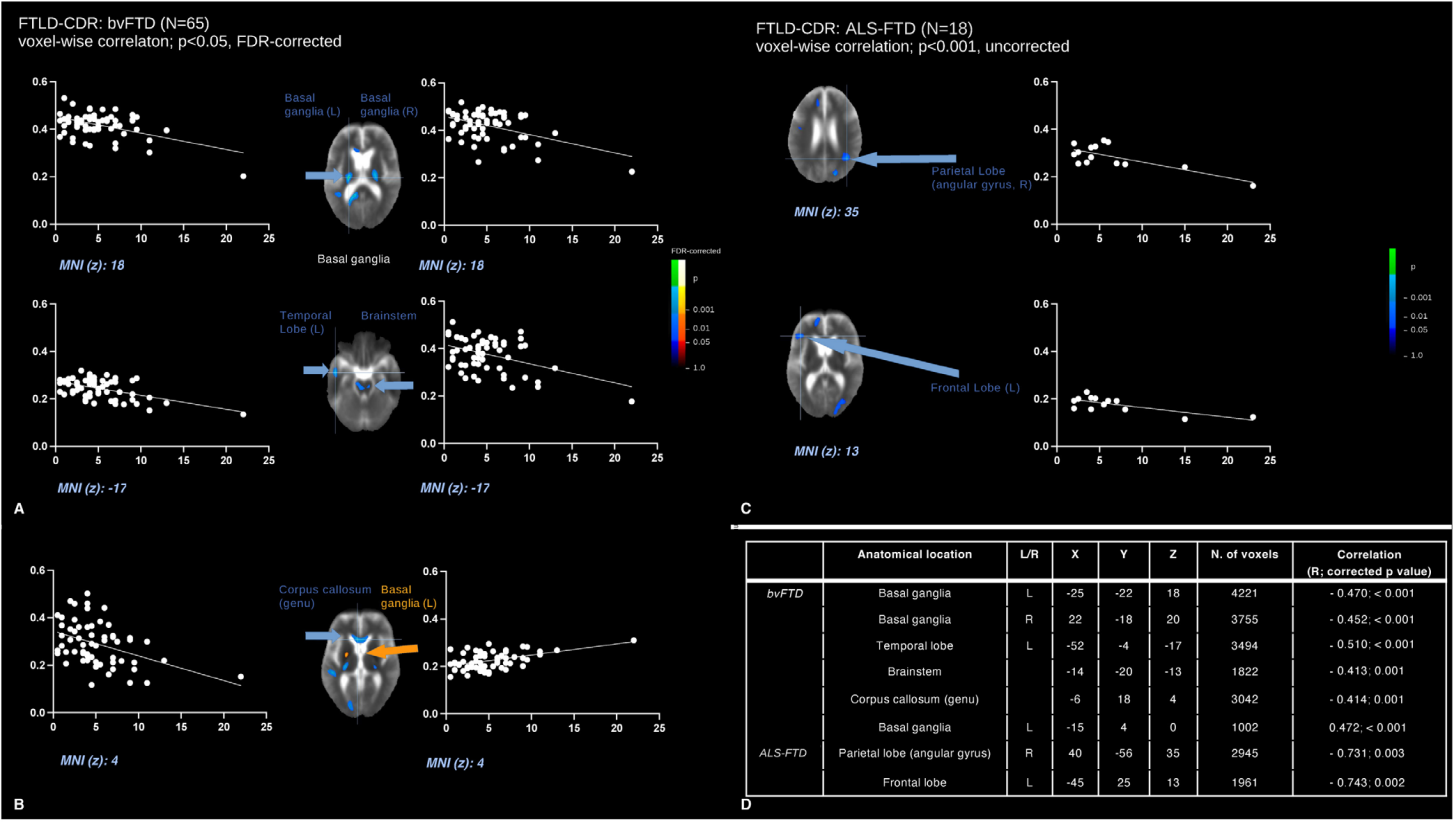
Whole‐brain‐based spatial statistics (WBSS) for correlation of voxel‐wise FA values with clinical scores. This figure displays slice‐wise views of cross‐sectional correlations of fractional anisotropy (FA, *y* axis) in 65 patients with bvFTD (A, B) and 18 patients with ALS‐FTD (C), respectively, with the sum of boxes of the frontotemporal lobar degeneration modified‐clinical dementia rating (FTLD‐CDR, *x* axis) score. Main clusters are shown with Montreal Neurological Institute [MNI] coordinates of the peak voxel. The significance level is indicated by the color bar: Cool colors represent a negative correlation while warm colors represent a positive correlation. Corrections for multiple comparisons were applied using the false discovery rate at *p* < 0.05 and a cluster‐size approach. Details for each cluster (correlation coefficient *R* and corrected *p* value, as well as size, expressed as n. of voxels) are displayed in the lower‐right quadrant (D). ALS‐FTD, amyotrophic lateral sclerosis associated with frontotemporal dementia; bvFTD, behavioral variant of frontotemporal dementia; L/R, left/right; MNI, Montreal Neurological Institute; WBSS, whole brain spatial statistics.

A voxel‐wise correlation (Figure 3A,B) of FA maps from 65 patients with bvFTD with their score of disease progression (sum of boxes of the frontotemporal lobar degeneration modified‐clinical dementia rating, FTLD‐CDR) revealed significant negative correlations in the bilateral basal ganglia. WM of the left temporal lobe, genu of the CC, and brainstem WM. Additionally, a cluster in the left basal ganglia was involved in a positive correlation.

A voxel‐wise correlation analysis of FA maps from 18 patients with ALS‐FTD with the sum of boxes of the FTLD‐CDR score (Figure 3C) revealed significant negative correlations in the WM of the right parietal lobe and the left frontal lobe.

When correlating ABV data with the sum of boxes of the FTLD‐CDR score, significant inverse correlations were found in bvFTD patients for a series of SOIs including the insula, amygdala, striatum, and the frontal lobe (FDR‐corrected). Differently, only uncorrected correlations could be observed in these patients by ABV for the right insula and the bilateral caudate (Table 4A,B).

**TABLE 4:** Correlation of FTLDCDR scores with volumetric values at SOIs level.

A	Anatomical location	*p* (FDR‐corrected)	*R*
bvFTD	Left insula	0.023	−0.445
	Cerebrum GM	0.023	−0.398
	Left amygdala	0.046	−0.350
	Left caudate	0.046	−0.335
	Right amygdala	0.046	−0.330
	Right insula	0.046	−0.332
	Left thalamus	0.046	−0.324
	Right caudate	0.046	−0.316
	Left putamen	0.046	−0.311
	Left frontal lobe	0.046	−0.309

B	Anatomical location	*p* (uncorrected)	*R*
ALS‐FTD	Right insula	0.046	−0.585
	Left caudate	0.036	−0.608
	Right caudate	0.049	−0.579

*Note:* This table displays *p* values and *R* coefficients for correlations of volumes in 65 patients with bvFTD (A) and 18 patients with ALS‐FTD (B), respectively, with the sum of boxes of the frontotemporal lobar degeneration modified‐clinical dementia rating (FTLD‐CDR) score. Corrections for multiple comparisons were applied for bvFTD with the false discovery rate approach; no correlation survived correction for ALS‐FTD. For this reason, uncorrected *p* values are listed.

Abbreviations: ALS‐FTD, amyotrophic lateral sclerosis associated with frontotemporal dementia; bvFTD, behavioral variant of frontotemporal dementia; FDR, false discovery rate; GM, Gray matter.

### 3.8 Random Forest

**FIGURE 4:**
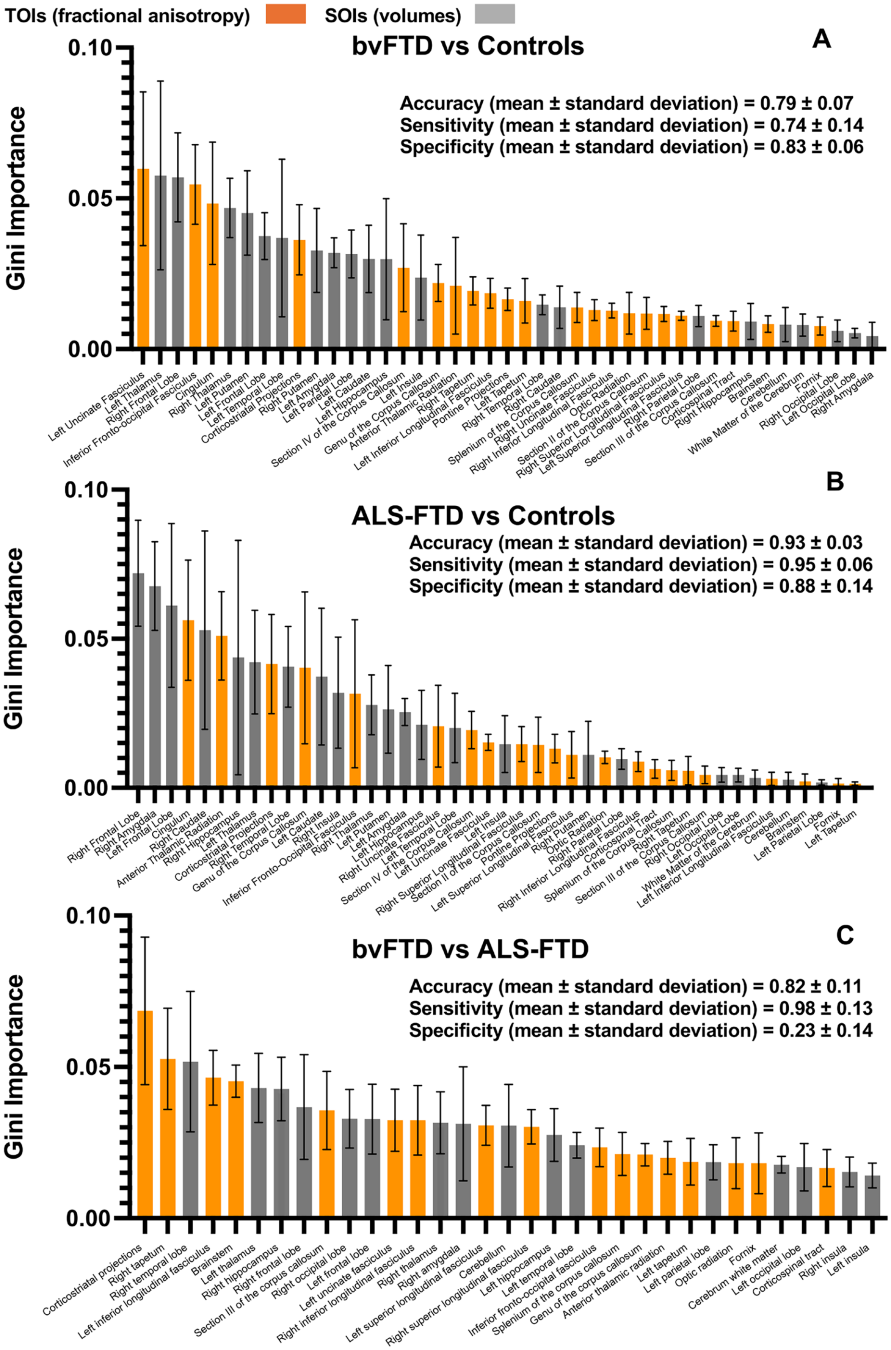
Classification of patients into diagnostic groups with a random forest algorithm. This illustration shows the Gini importance values derived from the random forest approach for classifying bvFTD (A) and ALS‐FTD (B) patients versus controls as well as patients with bvFTD versus patients with ALS‐FTD (C), using TOIs from TFAS and SOIs from ABV, as well as the averaged values for accuracy, sensitivity, and specificity between the 5 fold‐calculations for each comparison. ALS‐FTD, amyotrophic lateral sclerosis associated with frontotemporal dementia; bvFTD, behavioral variant of frontotemporal dementia; SOI, structures of interest; TOIs, tracts of interest.

A Random Forest model was employed to classify bvFTD and ALS‐FTD patients against controls, as well as bvFTD against ALS‐FTD patients, incorporating all the TOIs and SOIs analyzed in the study. Averaged accuracy, sensitivity and specificity of the models, as well as the Gini Importance for the used features, are summarized in Figure 4A–C. The Corticostriatal projections, frontal lobe and temporal lobe were important SOIs/TOIs, consistently ranked among the most important 10 features across the used models.

## 4 Discussion

Our combined voxel‐wise DTI and ABV analyses revealed marked white matter degeneration in the uncinate fasciculus, corpus callosum, and inferior longitudinal fasciculus, alongside gray matter atrophy in the frontal and temporal lobes, insula, and limbic structures, in both bvFTD and ALS‐FTD patients compared to controls. Our findings align with previous studies [23, 47, 48, 49, 50, 51], demonstrating FA reduction and atrophy in both bvFTD [19, 47, 49, 50, 52] and ALS‐FTD [51] in large regions of the frontal and temporal lobes as well as, for ALS‐FTD, in the motor cortex/CST [5, 51]. These findings were complemented by the TFAS analysis which highlighted a reduction in FA in the UF, CC, and cingulum bundle in both bvFTD and ALS‐FTD, in agreement with previous tract‐based reports [23, 48, 52].

ABV allowed us to address the relationship between WM and GM alterations in the ALS‐FTD continuum. The concept of a coupling between WM and GM damage in bvFTD has been previously undermined, with some studies reporting widespread WM damage relative to restricted cortical atrophy in the frontal and temporal lobes [48, 50]. Leveraging fine‐grained volumetric data, we identified a spatial gradient of GM atrophy, most pronounced in frontal, temporal, and limbic subcortical regions and comparatively less in parieto‐occipital areas, as reflected by volumetric *z*‐scores (Table 3A). This topography corresponded well with the TFAS results which revealed marked involvement of the UF (highly significant findings in both the bvFTD and ALS‐FTD groups), linking frontal and temporal cortices with limbic hubs [53], while showing relatively milder changes in the SLF (trend‐level significance in the bvFTD group; nonsignificant for the ALS‐FTD group) as a tract that connects anterior regions with more posterior parietal and occipital cortices [54].

Longitudinal WBSS and TFAS analyses (Figure 2A–D) in bvFTD revealed a predominantly antero–posterior trajectory of WM degeneration, with marked progression along the CC and ILF. In parallel, GM volumetric decline was most pronounced in the left temporal lobe, consistent with ILF involvement, in the bilateral insula, in line with UF involvement, and in subcortical regions such as the cerebellum, hippocampi, left amygdala, and bilateral caudate and putamen (Table 3B). Interestingly, the robust progression of WM degeneration in the CC was accompanied by only minor volumetric reductions in parietal lobes.

A decoupling of the localization of longitudinal GM and WM changes, with WM alterations both preceding and exceeding GM in magnitude, has been described in symptomatic FTLD cases [19, 48, 50, 55]. This supports our findings and makes it less likely that they are exclusively explained by dropout bias, whereby only milder patients with WM‐predominant, earlier‐stage damage would remain in the cohort while those with more advanced GM atrophy discontinue follow‐up. A definitive assessment of potential effects of dropout bias is inherently difficult and would remain speculative given the limited sample size and the multifactorial reasons for attrition in longitudinal neurodegenerative cohorts [56]. While a direct comparison of the magnitude of change between DTI and ABV would certainly be of interest, the two methods rely on fundamentally different principles. As such, a straightforward comparison based on normalized *z*‐scores would provide only a partial picture. In the present study, we therefore regard these techniques as complementary, offering converging but distinct perspectives on disease progression rather than serving as direct tests of relative sensitivity, that can be explored in future research.

Nevertheless, we acknowledge that the possible inclusion of genetic cases, whose temporal sequence of WM and GM alterations differs from sporadic FTLD [57, 58, 59], could have influenced the observed pattern. For example, in *GRN* and *MAPT* presymptomatic carriers WM tract degeneration has been reported as an early target of degeneration in the UF. However, even in such cases, WM and GM damage generally co‐occur in the symptomatic stage [57]. A relatively mild [15, 16, 60, 61] and slowly progressing [15] atrophy, characterized by a predominantly frontal rather than temporal epicenter of GM alterations [15, 60], has previously been reported also in bvFTD associated with a *C9orf72* mutation and in presymptomatic *C9orf72* carriers [62]. The low rate of progression especially aligns with that reported in our sample of bvFTD patients, whose genetic status is unknown. Considering the relatively high prevalence of *C9orf72* mutations among bvFTD cases [12, 13], it is plausible that, if genetic cases were present, they most likely involved *C9orf72*. Furthermore, the distinct trajectories of GM alterations associated with different mutations, such as those in the *MAPT* gene (predominantly focal anterior and medial temporal atrophy [60]) or *GRN* (typically asymmetric with greater parieto‐temporal involvement [60]) would likely have markedly influenced our results if they were predominant in our sample. Overall, we cannot fully rule out that the relatively slow degree of longitudinal volumetric reductions reported could be partially attributed to the inclusion of genetic cases, most likely *C9orf72* carriers. This limits the generalizability of our findings. Future research should aim to balance sufficiently large cohorts with detailed genetic characterization to disentangle these mutation‐specific patterns.

A secondary objective of this study was to determine whether alterations in TOIs and SOIs in ALS‐FTD parallel those typically seen in bvFTD. ALS‐FTD patients were confirmed to exhibit WM and GM features that mirrored those of bvFTD. Specifically, our findings were consistent with previous studies in demonstrating that the WM microstructural alterations [51, 63] and GM atrophy [5, 51] of bvFTD and ALS‐FTD patients overlapped in the frontotemporal lobes. Despite the overall similarities, our findings also highlight syndrome‐specific features in ALS‐FTD. Specifically, the DTI‐WBSS analysis showed preferential involvement of the upper CST (Figure S2A,B), echoing the signature of structural alterations in ALS [32, 64]. This “frontotemporal‐plus motor” pattern of structural damage for ALS‐FTD supports its relative proximity to bvFTD, suggesting it represents a distinct TDP43‐related phenotype rather than a late‐stage evolution from purely motor ALS [5], whose functional and structural alterations tend to remain limited to motor networks in the absence of cognitive and behavioral impairment [65].

Furthermore, ABV analysis demonstrated more marked volumetric reductions in subcortical SOIs for ALS‐FTD, particularly the amygdala, hippocampus, and thalamus (Table 3A). Subcortical [26, 61, 66, 67] involvement has been previously reported in the presence of *C9orf72‐*positive status [26, 61, 66, 67]. Our result is consistent with these previous studies. As in our bvFTD cohort (and with similar limitations noted above and in the “Limitations” section), given the high prevalence of *C9orf72* carriers among ALS‐FTD patients [15, 16], it is possible that the pronounced thalamic atrophy observed in our cohort of unknown genetic status may in part be driven by the presence of *C9orf72* cases.

The interpretation of the higher volumetric reductions in the hippocampi and amygdala in our ALS‐FTD cohort than in the bvFTD group is more challenging. The most straightforward explanation is that this effect may reflect the relatively more homogeneous TDP‐43 pathology driving ALS‐FTD [8], in contrast to the more heterogeneous pathological spectrum of the bvFTD group, where the impact of TDP‐43 could be attenuated by the inclusion of patients with FTLD‐tau [10]. Hippocampal sclerosis is common in FTLD due to TDP‐43 [68], especially in the case of TDP‐43 type C [40, 69, 70], which underlies the semantic variant of the primary progressive aphasia [69]. By contrast, it has been associated less frequently with a *C9orf72* mutation [16] and when motor neurons are involved in FTLD on the neuropathological level [68, 71]. However, the latter neuropathological studies [68, 71] may not fully reflect the conditions relevant to our comparison, as a substantial portion of these patients with a neuropathological definition of FTLD and motor neuron involvement were clinically classified as bvFTD. In contrast, the involvement of hippocampal hubs by TDP‐43 has been described in neuropathological stage IV of ALS [64]. Furthermore, a previous ABV study has detected lower volumes in *C9orf72*‐positive than in sporadic ALS [72], contrarily to what has been reported in *C9orf72*‐positive ALS‐FTD [16]. In our study, the hippocampal and amygdalar atrophy we observed, though evident, was less pronounced than that reported in a parallel study by our group on the semantic variant of primary progressive aphasia [40]. Taken together, these findings suggest that ALS‐FTD, driven by TDP‐43 type A and B [8], may present an intermediate degree of limbic hub degeneration, more pronounced than that typically associated with FTLD‐tau pathology [73], but less pronounced than the degeneration linked to TDP‐43 type C in semantic variant primary progressive aphasia, with a possible modulation by *C9orf72*. Clarifying this hypothesis will require further studies incorporating genetic and neuropathological information.

To further test the robustness of our unbiased analysis we employed a Random Forest model to differentiate patients into their respective diagnostic categories using TFAS‐based FA metrics and ABV‐based volumetric data (Figure 4A–C). The results demonstrated a strong overlap between the regions that reached the highest statistical significance in patient‐control comparisons, reinforcing the importance of these areas in capturing disease‐related changes. In detail, the most influential features for group classification included frontal lobe TOIs and SOIs for both bvFTD and ALS‐FTD, further emphasizing the shared pattern of frontal structural alterations across these syndromes [51]. Subcortical SOIs were also among the top‐ranked features, with the thalami playing a key role in the bvFTD algorithm and the hippocampi being particularly important in the ALS‐FTD algorithm. Importantly, we could demonstrate that, despite being trained with a comparatively small number of individual datasets, a random forest model achieved a moderately good degree of accuracy when it was instructed to classify bvFTD against ALS‐FTD.

Our longitudinal FA‐clinical correlation analysis suggests that, in bvFTD, disease progression was primarily associated with degeneration in the WM of subcortical structures, temporal WM, basal ganglia, and the CC. One of the correlations observed in the basal ganglia was positive; however, this is unlikely to reflect genuine structural preservation. Instead, it most likely represents a pseudo‐increase in FA due to DTI's known limitations in regions with complex fiber architecture [23, 24]. In such contexts, selective degeneration of one fiber population (in our case, potentially involving corticostriatal projections) while intersecting fibers remain relatively preserved can spuriously elevate FA values. This phenomenon was previously reported in other neurodegenerative conditions, where spared motor‐related projection fibers produced higher FA signals as they crossed affected association fibers within the SLF [74]. Future studies should address this issue using advanced models such as NODDI, which disentangle neurite density from orientation dispersion [75].

In contrast, in ALS‐FTD, the decline appeared to be driven primarily by degeneration in the WM of neocortical regions, particularly the parietal and frontal lobes. Correlations with volumetric data (Table 4A,B) further support the clinical significance of the changes revealed by comparison with controls in the frontal lobes, insula, amygdala for bvFTD and in the basal ganglia for both syndromes.

These findings align with the notion established by both pathological [64, 76] and DTI [24, 32, 77] studies that TDP‐43 pathology in ALS progresses in a corticofugal pattern along the sensorimotor network, extending downward from the cortex toward the brainstem.

Our results have to be considered in the context of several limitations. First, longitudinal analyses were conducted only in bvFTD patients, as longitudinal data for ALS‐FTD were not available. In addition, we were unable to include a cohort of purely motor ALS patients. This limits our ability to directly track and compare the longitudinal trajectories of WM and GM alterations in ALS with and without cognitive/behavioral involvement. As a result, we cannot conclusively determine whether ALS‐FTD differs from ALS from the outset, or whether it may emerge as a later disease stage. Nonetheless, previous studies have reported structural and functional differences between ALS and ALS‐FTD [5]. The consistency of our findings with these previous investigations reinforces the hypothesis of ALS‐FTD as a distinct phenotypic manifestation within the TDP‐43 spectrum. Second, beyond the absence of neuropathological confirmation, our study also lacked genetic data, particularly *C9orf72* status, which would have been crucial to corroborate whether the observed effects were mediated by TDP‐43 in both bvFTD and ALS‐FTD. Third, a more detailed cognitive and behavioral profiling in addition to the core diagnostic and functional assessments included would have been valuable to further characterize and differentiate the bvFTD and ALS‐FTD groups as well as to correlate the alterations in their WM and GM profiles to the differences in their clinical picture. Future studies incorporating a broader range of clinical measures will be important to complement and extend our findings. Fourth, despite the relatively modest longitudinal sample size, our use of standardized DTI acquisition protocols, validated for reliability and reproducibility, ensures that our results remain robust and generalizable, even in limited cohorts [25, 33]. Fifth, from the methodological perspective, a significantly higher head motion in clinical cohorts in neurodegeneration compared to controls has to be considered. This might differentially impact the voxel‐wise analyses. However, in this study, we minimized the presence of such motion‐induced clusters among the results by a standardized quality control [34]. Finally, the limited availability of follow‐up MRI data may have favored the inclusion of patients with milder disease progression, introducing a dropout bias. This could have influenced our results leading us to underestimate the degree of progression in WM and GM changes.

In conclusion, our study highlights the complementary value of DTI and ABV in capturing both structural and microstructural brain alterations associated with bvFTD and ALS‐FTD and in tracking disease progression over time. Our findings suggest that, within the bvFTD spectrum, neurodegeneration tends to spread along axonal pathways linking functionally connected brain regions, rather than progressing merely through anatomical proximity between adjacent but functionally unrelated areas. Furthermore, our complementary unbiased and tract‐based approaches strengthen the hypothesis that ALS‐FTD stands as a distinct phenotype within the TDP‐43 clinicopathological spectrum, aligning more closely, from the perspective of brain structural changes, with bvFTD than with purely motor ALS.

Although the attempt to provide clinico‐pathological correlations remains speculative, it highlights the potential of structural MRI not only for phenotypic classification but also for probing disease mechanisms. Future studies incorporating longitudinal and pathology‐confirmed cohorts will be critical to further clarify these associations.

## Author Contributions

Conceptualization: Marco Michelutti, Hans‐Peter Müller, and Jan Kassubek; Methodology: Marco Michelutti, Hans‐Jürgen Huppertz, Heiko Volkmann, Hans‐Peter Müller, and Jan Kassubek; Software: Hans‐Jürgen Huppertz and Hans‐Peter Müller; Data curation: Marco Michelutti, Sarah Anderl‐Straub, Heiko Volkmann, Angela Rosenbohm, Albert C. Ludolph, Markus Otto, Hans‐Peter Müller, and Jan Kassubek; Investigation: Marco Michelutti, Hans‐Peter Müller, and Jan Kassubek; Validation: Marco Michelutti, Hans‐Jürgen Huppertz, Hans‐Peter Müller, and Jan Kassubek; Formal analysis: Marco Michelutti, Hans‐Jürgen Huppertz, Heiko Volkmann, Hans‐Peter Müller, and Jan Kassubek; Supervision: Hans‐Jürgen Huppertz, Giancarlo Logroscino, Hans‐Peter Müller, and Jan Kassubek; Visualization: Marco Michelutti and Hans‐Peter Müller; Project administration: Hans‐Peter Müller and Jan Kassubek; Resources: Sarah Anderl‐Straub, Paolo Manganotti, Angela Rosenbohm, Albert C. Ludolph, and Markus Otto; Writing – original draft: Marco Michelutti; Writing – review and editing: Marco Michelutti, Hans‐Jürgen Huppertz, Heiko Volkmann, Daniele Urso, Benedetta Tafuri, Salvatore Nigro, Hans‐Peter Müller, and Jan Kassubek.

## Funding

The authors have nothing to report.

## Conflicts of Interest

Prof. Jan Kassubek serves as the Specialty Chief Editor of Applied Neuroimaging for Frontiers in Neurology. This role had no influence on the editorial process or peer review of this manuscript. The authors declare no other competing interests.

## Supporting information


**Figure S1:** Whole brain differences in Fractional Anistropy between subjects with Subjective Cognitive Decline and Healthy Controls.
**Figure S2:** Whole‐brain‐based spatial statistics (WBSS) and Tract‐of‐Interest (TOI)‐based statistics for cross‐sectional comparison of involvement of the corticospinal tract (CST) in the FA maps of patients with bvFTD and ALS‐FTD versus controls.
**Figure S3:** Whole‐brain‐based spatial statistics for cross‐sectional comparison of MD maps of patients with bvFTD and patients with ALS‐FTD versus controls.
**Figure S4:** Schematic example of the DTI and ABV processing pipelines.
**Table S1:** Cohort characteristics and MRI acquisition protocols across study sites.
**Table S2:** Cross sectional differences in the TOIs between healthy controls and participants with subjective cognitive decline.
**Table S3:** Cross sectional differences in the SOIs between healthy controls and participants with subjective cognitive decline.
**Table S4:** Whole‐brain‐based spatial statistics for cross‐sectional comparison of FA maps of patients with bvFTD (*N* = 65) and patients with ALS‐FTD (*N* = 18) versus controls (*N* = 39).
**Table S5:** Cross‐sectional mean diffusivity (MD) differences in the white matter TOIs at group‐level.
**Table S6:** Whole‐brain‐based spatial statistics for cross‐sectional comparison of FA maps of patients with bvFTD with available longitudinal data (*N* = 19) versus controls (*N* = 39).

## Data Availability

The data that support the findings of this study are available on request from the corresponding author. The data are not publicly available due to privacy or ethical restrictions.
