# Supplementary material for: MRI‐DTI Biomarkers Along the Continuum of Behavioral Variant Frontotemporal Dementia
Source: Eur J Neurol. 2025 Nov 30;32(12):e70438. doi: 10.1111/ene.70438 (PMC12665338; doi:10.1111/ene.70438)
Supplement: Supplementary file 1 — Figure S1: Whole brain differences in Fractional Anistropy between subjects with Subjective Cognitive Decline and Healthy Controls. Figure S2: Whole‐brain‐based spatial statistics (WBSS) and Tract‐of‐Interest (TOI)‐based statistics for cross‐sectional comparison of involvement of the corticospinal tract (CST) in the FA maps of patients with bvFTD and ALS‐FTD versus controls. Figure S3: Whole‐brain‐based spatial statistics for cross‐sectional comparison of MD maps of patients with bvFTD and patients with ALS‐FTD versus controls. Figure S4: Schematic example of the DTI and ABV processing pipelines. Table S1: Cohort characteristics and MRI acquisition protocols across study sites. Table S2: Cross sectional differences in the TOIs between healthy controls and participants with subjective cognitive decline. Table S3: Cross sectional differences in the SOIs between healthy controls and participants with subjective cognitive decline. Table S4: Whole‐brain‐based spatial statistics for cross‐sectional comparison of FA maps of patients with bvFTD (N = 65) and patients with ALS‐FTD (N = 18) versus controls (N = 39). Table S5: Cross‐sectional mean diffusivity (MD) differences in the white matter TOIs at group‐level. Table S6: Whole‐brain‐based spatial statistics for cross‐sectional comparison of FA maps of patients with bvFTD with available longitudinal data (N = 19) versus controls (N = 39). [file ENE-32-e70438-s001.docx › ene70438-sup-0007-TableS2@SupplementaryTable2 .docx]

**Supplementary Table 2** Cross sectional differences in the TOIs between healthy controls and participants with subjective cognitive decline

| **Tract of interest (TOI) analysis** | **Healthy controls (HC)** | | **Participants with subjective cognitive decline (SCD)** | | **HC vs SCD (p value )** |
| --- | --- | --- | --- | --- | --- |
|  | **Mean** | **SD** | **Mean** | **SD** |  |
| Left uncinate fasciculus | 0.30 | 0.02 | 0.29 | 0.02 | ns |
| Right uncinate fasciculus | 0.29 | 0.03 | 0.27 | 0.02 | ns |
| Genu of the corpus callosum | 0.37 | 0.05 | 0.33 | 0.05 | ns |
| Splenium of the corpus callosum | 0.37 | 0.05 | 0.36 | 0.05 | ns |
| Section II of the corpus callosum | 0.28 | 0.02 | 0.28 | 0.02 | ns |
| Section III of the corpus callosum | 0.31 | 0.05 | 0.30 | 0.03 | ns |
| Section IV of the corpus callosum | 0.37 | 0.07 | 0.35 | 0.03 | ns |
| Left superior longitudinal fasciculus | 0.35 | 0.04 | 0.35 | 0.02 | ns |
| Right superior longitudinal fasciculus | 0.39 | 0.04 | 0.38 | 0.03 | ns |
| Left inferior longitudinal fasciculus | 0.32 | 0.03 | 0.31 | 0.03 | ns |
| Right inferiori longitudinal fasciculus | 0.33 | 0.04 | 0.31 | 0.03 | ns |
| Inferior fronto-occipitalis fasciculus | 0.30 | 0.03 | 0.30 | 0.02 | ns |
| Cingulum | 0.39 | 0.05 | 0.38 | 0.03 | ns |
| Pontine projections | 0.37 | 0.03 | 0.35 | 0.02 | ns |
| Anterior thalamic radiation | 0.29 | 0.03 | 0.28 | 0.02 | ns |
| Corticostriatal projections | 0.29 | 0.03 | 0.29 | 0.02 | ns |
| Corticospinal tract | 0.38 | 0.03 | 0.37 | 0.03 | ns |
| Optic radiation | 0.37 | 0.04 | 0.37 | 0.03 | ns |
| Fornix | 0.27 | 0.04 | 0.27 | 0.02 | ns |
| Left tapetum | 0.34 | 0.06 | 0.33 | 0.02 | ns |
| Right tapetum | 0.38 | 0.05 | 0.37 | 0.03 | ns |

This table shows the differences in mean fractional anisotropy (FA) values at baseline between 10 healthy controls and 29 participants with subjective cognitive decline. No significant difference could be detected after correction for multiple comparison. Additionaly. mean and standard deviation are reported for each TOI for documentation purposes.

**Legend** TOIs: Tracts of Interest; SD: Standard Deviation.
