# Supplementary material for: MRI‐DTI Biomarkers Along the Continuum of Behavioral Variant Frontotemporal Dementia
Source: Eur J Neurol. 2025 Nov 30;32(12):e70438. doi: 10.1111/ene.70438 (PMC12665338; doi:10.1111/ene.70438)
Supplement: Supplementary file 1 — Figure S1: Whole brain differences in Fractional Anistropy between subjects with Subjective Cognitive Decline and Healthy Controls. Figure S2: Whole‐brain‐based spatial statistics (WBSS) and Tract‐of‐Interest (TOI)‐based statistics for cross‐sectional comparison of involvement of the corticospinal tract (CST) in the FA maps of patients with bvFTD and ALS‐FTD versus controls. Figure S3: Whole‐brain‐based spatial statistics for cross‐sectional comparison of MD maps of patients with bvFTD and patients with ALS‐FTD versus controls. Figure S4: Schematic example of the DTI and ABV processing pipelines. Table S1: Cohort characteristics and MRI acquisition protocols across study sites. Table S2: Cross sectional differences in the TOIs between healthy controls and participants with subjective cognitive decline. Table S3: Cross sectional differences in the SOIs between healthy controls and participants with subjective cognitive decline. Table S4: Whole‐brain‐based spatial statistics for cross‐sectional comparison of FA maps of patients with bvFTD (N = 65) and patients with ALS‐FTD (N = 18) versus controls (N = 39). Table S5: Cross‐sectional mean diffusivity (MD) differences in the white matter TOIs at group‐level. Table S6: Whole‐brain‐based spatial statistics for cross‐sectional comparison of FA maps of patients with bvFTD with available longitudinal data (N = 19) versus controls (N = 39). [file ENE-32-e70438-s001.docx › ene70438-sup-0009-TableS4@SupplementaryTable4 .docx]

**Supplementary Table 4** Whole-brain-based spatial statistics for cross-sectional comparison of FA maps of patients with bvFTD (N=65) and patients with ALS-FTD (N=18) vs controls (N=39)

|  | **Anatomical location** | **L/R** | **X** | **Y** | **Z** | **N. of voxels** |
| --- | --- | --- | --- | --- | --- | --- |
| **bvFTD** | Fronto-temporal lobes | L/R | ±18 | 24 | 26 | 220961 |
| **ALS-FTD** | Fronto-temporal lobes | L/R | ±18 | 24 | 26 | 217790 |

This table shows details of the clusters where FA changes were detected in patients with bvFTD (n=65) and ALS-FTD (N=18) compared to controls (N=39) by whole-brain based spatial statistics (**figure 1**).

**Legend** FA: Fractional anisotropy**;** bvFTD: behavioral variant of frontotemporal dementia; ALS-FTD: amyotrophic lateral sclerosis associated with frontotemporal dementia; L/R: left/right.
