# Supplementary material for: MRI‐DTI Biomarkers Along the Continuum of Behavioral Variant Frontotemporal Dementia
Source: Eur J Neurol. 2025 Nov 30;32(12):e70438. doi: 10.1111/ene.70438 (PMC12665338; doi:10.1111/ene.70438)
Supplement: Supplementary file 1 — Figure S1: Whole brain differences in Fractional Anistropy between subjects with Subjective Cognitive Decline and Healthy Controls. Figure S2: Whole‐brain‐based spatial statistics (WBSS) and Tract‐of‐Interest (TOI)‐based statistics for cross‐sectional comparison of involvement of the corticospinal tract (CST) in the FA maps of patients with bvFTD and ALS‐FTD versus controls. Figure S3: Whole‐brain‐based spatial statistics for cross‐sectional comparison of MD maps of patients with bvFTD and patients with ALS‐FTD versus controls. Figure S4: Schematic example of the DTI and ABV processing pipelines. Table S1: Cohort characteristics and MRI acquisition protocols across study sites. Table S2: Cross sectional differences in the TOIs between healthy controls and participants with subjective cognitive decline. Table S3: Cross sectional differences in the SOIs between healthy controls and participants with subjective cognitive decline. Table S4: Whole‐brain‐based spatial statistics for cross‐sectional comparison of FA maps of patients with bvFTD (N = 65) and patients with ALS‐FTD (N = 18) versus controls (N = 39). Table S5: Cross‐sectional mean diffusivity (MD) differences in the white matter TOIs at group‐level. Table S6: Whole‐brain‐based spatial statistics for cross‐sectional comparison of FA maps of patients with bvFTD with available longitudinal data (N = 19) versus controls (N = 39). [file ENE-32-e70438-s001.docx › ene70438-sup-0010-TableS5@SupplementaryTable5 .docx]

**Supplementary Table 5** Cross-sectional mean diffusivity (MD) differences in the white matter TOIs at group-level.

| **Tract of interest (TOI) analysis** |  | **bvFTD** | | |  | **ALS-FTD** | | |  | **Controls** | |
| --- | --- | --- | --- | --- | --- | --- | --- | --- | --- | --- | --- |
|  | **Mean** | **St. Dev.** | **z score bvFTD vs Controls** | **p value** | **Mean** | **St. Dev.** | **z score ALS-FTD vs Controls** | **p value** | **z score ALS-FTD vs bvFTD** | **Mean** | **St. Dev.** |
| Left uncinate fasciculus | 0.34 | 0.05 | -1.27 | < 0.001 | 0.37 | 0.05 | -2.38 | < 0.001 | -0.49 | 0.29 | 0.02 |
| Right uncinate fasciculus | 0.33 | 0.05 | -1.06 | < 0.001 | 0.37 | 0.04 | -3.05 | < 0.001 | -0.80 | 0.29 | 0.02 |
| Genu of the corpus callosum | 0.46 | 0.10 | -1.15 | < 0.001 | 0.48 | 0.08 | -1.76 | < 0.001 | -0.22 | 0.37 | 0.06 |
| Splenium of the corpus callosum | 0.47 | 0.11 | -0.95 | < 0.001 | 0.48 | 0.11 | -1.24 | < 0.001 | -0.07 | 0.38 | 0.07 |
| Section II of the corpus callosum | 0.51 | 0.09 | -1.25 | < 0.001 | 0.54 | 0.09 | -1.98 | < 0.001 | -0.31 | 0.41 | 0.05 |
| Section III of the corpus callosum | 0.52 | 0.09 | -0.85 | < 0.001 | 0.53 | 0.08 | -1.12 | < 0.001 | -0.07 | 0.45 | 0.06 |
| Section IV of the corpus callosum | 0.46 | 0.09 | -0.95 | < 0.001 | 0.46 | 0.09 | -1.10 | 0.004 | -0.02 | 0.38 | 0.06 |
| Left superior longitudinal fasciculus | 0.31 | 0.05 | -0.99 | < 0.001 | 0.32 | 0.04 | -1.60 | < 0.001 | -0.17 | 0.27 | 0.02 |
| Right superior longitudinal fasciculus | 0.30 | 0.04 | -0.83 | < 0.001 | 0.31 | 0.03 | -1.89 | < 0.001 | -0.43 | 0.27 | 0.02 |
| Left inferior longitudinal fasciculus | 0.30 | 0.05 | -0.86 | < 0.001 | 0.31 | 0.03 | -1.73 | < 0.001 | -0.20 | 0.27 | 0.02 |
| Right inferior longitudinal fasciculus | 0.29 | 0.04 | -0.74 | < 0.001 | 0.30 | 0.03 | -1.58 | < 0.001 | -0.30 | 0.27 | 0.02 |
| Inferior fronto-occipitalis fasciculus | 0.40 | 0.07 | -1.28 | < 0.001 | 0.43 | 0.05 | -2.19 | < 0.001 | -0.36 | 0.33 | 0.04 |
| Cingulum | 0.50 | 0.10 | -0.92 | < 0.001 | 0.52 | 0.10 | -1.28 | < 0.001 | -0.11 | 0.42 | 0.06 |
| Pons/brainstem | 0.41 | 0.07 | 0.01 | ns | 0.46 | 0.08 | -0.86 | 0.031 | -0.67 | 0.41 | 0.04 |
| Anterior thalamic radiation | 0.41 | 0.07 | -1.12 | < 0.001 | 0.47 | 0.08 | -2.57 | < 0.001 | -0.85 | 0.34 | 0.04 |
| Corticostriatal projections | 0.41 | 0.13 | -1.13 | < 0.001 | 0.47 | 0.14 | -1.94 | < 0.001 | -0.43 | 0.29 | 0.07 |
| Corticospinal tract | 0.31 | 0.04 | -0.74 | < 0.001 | 0.32 | 0.04 | -1.40 | < 0.001 | -0.40 | 0.29 | 0.03 |
| Optic radiation | 0.42 | 0.13 | -0.36 | ns | 0.43 | 0.15 | -0.49 | ns | -0.11 | 0.37 | 0.10 |
| Fornix | 0.57 | 0.06 | -0.87 | < 0.001 | 0.62 | 0.06 | -1.62 | < 0.001 | -0.70 | 0.52 | 0.06 |
| Left tapetum | 0.45 | 0.08 | -0.82 | < 0.001 | 0.46 | 0.06 | -1.19 | < 0.001 | -0.17 | 0.38 | 0.06 |
| Right tapetum | 0.33 | 0.06 | -0.63 | 0.001 | 0.35 | 0.05 | -1.19 | < 0.001 | -0.39 | 0.30 | 0.04 |

This table shows the differences in mean diffusivity (MD) values at baseline between 65 patients with bvFTD. 18 patients with ALS-FTD. and 39 controls. P values are reported if the difference between the mean volumetric and FA values of controls and patients with bvFTD and ALS-FTD. respectively. was significant (p < 0.05. corrected for multiple comparisons).

**Legend**: TOIs: Tracts of Interest; St. Dev: Standard Deviation; bvFTD: behavioral variant of frontotemporal dementia;ALS-FTD: amyotrophic lateral sclerosis associated with frontotemporal dementia; ns: non-significant.
